# Recreational Water Illness and Injury Prevention Week — May 19–25

**Published:** 2014-05-16

May 19–25, 2014, marks the 10th annual Recreational Water Illness and Injury Prevention Week. This observance highlights ways in which swimmers, parents, pool owners and operators, beach managers, and public health can maximize the health benefits of water-based physical activity, while avoiding water-associated illness and injury.

To help keep swimming a healthy and safe activity, CDC has recently published four reports on illness and injury risks associated with recreational water (e.g., pools and lakes) (1–4). CDC provides health and safety tips online to help swimmers, parents, and others prevent recreational water–associated illnesses (http://www.cdc.gov/healthywater/swimming/protection/triple-a-healthy-swimming.html); drownings (http://www.cdc.gov/homeandrecreationalsafety/water-safety/index.html); injury from pool chemicals (http://www.cdc.gov/healthywater/swimming/pools/preventing-pool-chemical-injuries.html); and exposure to harmful algal blooms (http://www.cdc.gov/healthcommunication/toolstemplates/entertainmented/tips/algalblooms.html).

CDC has also posted the second draft of the Model Aquatic Health Code (MAHC) (http://www.cdc.gov/mahc) for final public comment through May 27, 2014. MAHC guidelines for public treated recreational water venues (e.g., pools) are expected to be available this summer.
